# Editorial: The crosstalk between emerging cell death and immune microenvironment remodeling in cancer progression and treatment

**DOI:** 10.3389/fimmu.2025.1715186

**Published:** 2025-10-16

**Authors:** Bufu Tang, Xuanzhang Huang, Weiliang Hou, Jifeng Wang, Ling Wang

**Affiliations:** ^1^ Zhongshan Hospital, Fudan University, Shanghai, China; ^2^ The First Affiliated Hospital of China Medical University, Shenyang, China; ^3^ Shanghai Tenth People’s Hospital, Tongji University, Shanghai, China; ^4^ Vanderbilt University Medical Center, Nashville, TN, United States; ^5^ First Affiliated Hospital of Dalian Medical University, Dalian, China

**Keywords:** ICD, immune microenvironment, ferroptosis, cancer, RCD

## 1 Introduction

Cancer treatment is experiencing a fundamental transformation from conventional cytotoxic methods to precision-based immunotherapeutic strategies. Emerging forms of regulated cell death (RCD), including ferroptosis, autophagy, and immunogenic cell death (ICD), are intricately linked to the dynamic remodeling of the tumor immune microenvironment (TIME), presenting unprecedented opportunities for cancer treatment. An effective anti-cancer immune response requires precise orchestration of cellular and molecular mediators involved in the “cancer-immunity cycle” to prevent tumor progression and ultimately achieve eradication.

## 2 Summary of papers presented in this Research Topic

Among the numerous emerging RCD modalities, ferroptosis research demonstrates particular vibrancy. Xie et al. demonstrated through bibliometric analysis an exponential growth in ferroptosis research between 2014 and 2024, with a remarkable annual growth rate of 64.99%. The research focus is progressively shifting from basic mechanistic studies to clinical translation, particularly showcasing vast potential in overcoming drug resistance (Xie et al.). Echoing this trend, Ma et al. discovered that FXYD6 in ovarian cancer metastasis exerts tumor-suppressive effects by inducing ferroptosis and, utilizing machine learning approaches, developed a predictive model that provides novel targets and tools for precision therapy (Ma et al.). Autophagy, another significant RCD modality, exhibits a complex dual role in cancer progression. Jalali et al.‘s comprehensive review highlights that autophagy suppresses carcinogenesis in early-stage tumors by clearing damaged organelles; however, in advanced tumors, it may provide metabolic support for tumor cells, thereby promoting invasion, metastasis, and drug resistance (Jalali et al.). Consequently, autophagy presents a challenging therapeutic target, with the key to future research lying in its regulation within an appropriate “therapeutic window.” Furthermore, the strong coupling between autophagy and immune responses offers crucial insights for optimizing cellular therapies such as CAR-T. In the realm of precision delivery, innovative materials science is opening new avenues for tumor immunotherapy. The injectable immunotherapeutic hydrogel developed by Zhang et al. enables sustained local activation of the STING pathway following surgical resection in breast cancer, significantly enhancing dendritic cell maturation and T cell immune responses (Zhang et al.). In preclinical studies, this system achieved complete remission without recurrence for up to 120 days, demonstrating the powerful potential of localized immune delivery systems. Beyond host factors, the gut microbiome has been shown to significantly influence immunotherapy responses. Ding et al. revealed that butyrate, a metabolite of Fusobacterium nucleatum, alters colorectal cancer sensitivity to immunotherapy by activating the autophagy-lysosomal pathway and downregulating the DNA repair factor MLH1 (Ding et al.). In animal studies, the combination of butyrate with PD-L1 inhibitors demonstrated enhanced efficacy, suggesting that modulating the microbiome-immune axis could expand the population benefiting from immunotherapy.

Advances in multi-omics technologies are ushering in novel opportunities for precision cancer diagnosis and treatment. He et al. constructed a distant metastasis prediction model based on multi-omics data from lung cancer patient exosomes, identifying the sphingolipid metabolism pathway as a critical mediator of metastasis. The model, comprising 15 proteins, achieved an AUC of 0.95 in external validation, enabling early identification of high-risk patients. This achievement exemplifies the potential of multi-omics data in translational oncology. Metabolic pathway remodeling not only drives tumor growth but also profoundly impacts immune responses (He et al.). Ma et al. employed single-cell sequencing to reveal that programmed cell death (PCD)-related genes drive the immunosuppressive effects of the CEBPB^+^ fibroblast subpopulation in triple-negative breast cancer (TNBC) and are closely associated with poor prognosis. Their constructed prognostic model was validated in clinical samples and murine experiments, providing new avenues for improving TNBC immunotherapy (Ma et al.). Simultaneously, Han et al. concentrated their research efforts on disulfidptosis mechanisms, they demonstrated that this mechanism can predict endometrial cancer patients’ responses to immunotherapy and characterize immune environment features, this suggests that novel cell death modalities may serve as important reference criteria for individualized therapy approaches (Han et al.). Within clear cell renal cell carcinoma (ccRCC), ferroptosis exhibits complex functional characteristics, on one hand this mechanism can enhance anti-tumor immune responses through inducing cellular death, on the other hand it may accelerate tumor progression processes by excessively damaging immune cells. The deep understanding of ferroptosis and metabolic interactions explored by Chen et al. holds promise for driving the development processes of combination therapy regimens for renal cell carcinoma (He et al.). Additionally, Shan and Liu focused their attention on mucosal melanoma (MM) field, summarizing the potential value of immune checkpoint inhibitors, adoptive cell transfer therapy (ACT), anti-angiogenic treatments, and combination strategies in improving therapeutic effectiveness and overcoming drug resistance. This comprehensive methodology brings new hope for highly aggressive malignancies with historically limited effective treatment options (Shan and Liu). Yang et al. identified osteoporosis biomarkers associated with cellular death and mitochondrial function through multi-omics analysis and experimental validation methods. Their research revealed these molecules not only participate in disease progression processes, but also closely correlate with immune cell infiltration within immune microenvironments, thereby providing novel insights into bone-immune interactions and paving pathways for precision diagnostics and treatment approaches (Yang et al.). Concurrently, Xu et al. in their comprehensive review emphasized the intricate interplay relationships between ferroptosis and immune responses, indicating the synergistic potential of ferroptosis inducers combined with immune checkpoint inhibitors, thus providing theoretical foundations for future combination strategies (Xu et al.). In further translational research efforts, Chen et al. established a Regulated Cell Death-Related Index (RCDRI) using machine learning methods, this index consists of four genes (CD36, SERPINE1, TRIML2, GRP). The RCDRI demonstrated effectiveness in predicting prognosis and immunotherapy efficacy across multiple independent cohorts. Critically, experiments showed that TRIML2 knockdown significantly inhibited gastric cancer cell proliferation and migration, indicating that this index possesses both prognostic assessment and therapeutic guidance value (13).

## 3 Outlook

Despite significant recent advances, cancer therapy continues to face several formidable challenges: limitations in precise drug delivery systems, efficacy variations attributable to individual patient differences, and toxicity management issues associated with multi-drug combination regimens. Looking toward the future, the integration of artificial intelligence with single-cell multi-omics technologies will facilitate the mapping of increasingly refined molecular networks, enabling a paradigmatic transition from single-target modulation to comprehensive systems-based network intervention. Through the synergistic integration of cell death regulation, immune microenvironment remodeling, and metabolic reprogramming, we anticipate driving truly transformative breakthroughs in cancer treatment that will fundamentally reshape therapeutic outcomes for patients worldwide.

